# Evaluation of digital image analysis as a supportive tool for the stratification of head and neck vascular anomalies

**DOI:** 10.1007/s00405-020-06097-2

**Published:** 2020-06-02

**Authors:** Jovine Ehrenreich, Michael Bette, Ansgar Schmidt, Marion Roeßler, Udo Bakowsky, Urban W. Geisthoff, Boris A. Stuck, Robert Mandic

**Affiliations:** 1 Department of Otorhinolaryngology, Head and Neck Surgery, University Hospital Marburg, Philipps-Universität Marburg, 3. BA, +3/08070 Baldingerstrasse, 35033 Marburg, Germany; 2 Institute of Anatomy and Cell Biology, Philipps-Universität Marburg, Marburg, Germany; 3 Institute of Pathology, University Hospital Giessen and Marburg, Marburg, Germany; 4 Department of Pharmaceutical Technology and Biopharmacy, Philipps-Universität Marburg, Marburg, Germany

**Keywords:** Vascular anomaly, Head and neck, Stratification, Digital image analysis, Immunohistochemistry

## Abstract

**Background:**

The histological differentiation of individual types of vascular anomalies (VA), such as lymphatic malformations (LM), hemangioma (Hem), paraganglioma (PG), venous malformations (VeM), arteriovenous malformations (AVM), pyogenic granulomas (GP), and (not otherwise classified) vascular malformations (VM n.o.c.) is frequently difficult due to the heterogeneity of these anomalies. The aim of the study was to evaluate digital image analysis as a method for VA stratification

**Methods:**

A total of 40 VA tissues were examined immunohistologically using a selection of five vascular endothelial-associated markers (CD31, CD34, CLDN5, PDPN, VIM). The staining results were documented microscopically followed by digital image analyses based quantification of the candidate-marker-proteins using the open source program ImageJ/Fiji.

**Results:**

Differences in the expression patterns of the candidate proteins could be detected particularly when deploying the quotient of the quantified immunohistochemical signal values. Deploying signal marker quotients, LM could be fully distinguished from all other tested tissue types. GP achieved stratification from LM, Hem, VM, PG and AVM tissues, whereas Hem, PG, VM and AVM exhibited significantly different signal marker quotients compared with LM and GP tissues.

**Conclusion:**

Although stratification of different VA from each other was only achieved in part with the markers used, the results of this study strongly support the usefulness of digital image analysis for the stratification of VA. Against the background of upcoming new diagnostic techniques involving artificial intelligence and deep (machine) learning, our data serve as a paradigm of how digital evaluation methods can be deployed to support diagnostic decision making in the field of VAs.

## Introduction

Vascular anomalies (VA) of the head and neck area are a heterogeneous group of vascular diseases which can not only cause cosmetic but also life-threatening functional disorders, such as bleeding, dyspnea or dysphagia [1–3]. VA encompass vascular malformations such as venous (VeM), arteriovenous (AVM) and lymphatic malformations (LM) and paraganglioma (PG). Another subgroup are vascular tumors such as hemangioma (Hem) and pyogenic granuloma (GP). There are still many inconsistencies among the histopathological and clinical classifications of anomalies in each subgroup. This can lead to false diagnosis that negatively affects the choice of therapy. Currently, diagnosis of VA subtypes is typically performed by histopathological evaluation in conjunction with the clinical appearance of the VA [4]. In this context, digital quantification may help optimizing diagnosis thereby making it more objective. This prompted us to investigate whether immunohistological quantification of vascular anomaly tissues via digital image analysis allows a more accurate assignment into their different subtypes. For digital quantification we deployed the open source program ImageJ/Fiji which is used for scientific image analysis concerning numerous biological questions [5].

There are already well-established endothelial-associated markers such as CD34 and CD31 for vascular endothelia and Podoplanin (PDPN) for lymphatic endothelia. However, staining VA tissues with these markers does not ensure a distinct classification. Among the long list of other potential vascular markers are Claudin 5 (CLDN5) and Vimentin (VIM). CLDN5 is a transmembrane protein and structural component of tight-junctions and, therefore, takes part in establishing the paracellular barrier. It is expressed on epithelial as well as endothelial cells and recent studies show that it might be more than a “barrier-protein” as it seems to play a part in cell growth and transition from epithelial to mesenchymal tissue [6]. CLDN5 might be a possible marker for advanced VA, showing positive staining in both vascular and lymphatic endothelia but mostly in malignant anomalies and tumors [7]. This implies a role in the differentiation of VA.

VIM is an intermediate filament which is a structural component of the cytoskeleton serving to stabilize the integrity of cells and cohesion of tissues. In endothelial and vascular smooth muscle cells VIM is the major intermediate filament [8, 9]. A high VIM-expression marks the transition from epithelial to mesenchymal cells and was also seen in the progression and growth of different tumors [10, 11].

In the present study we used five endothelial-associated markers (CD34, CD31, VIM, PDPN, CLDN5) for immunohistological tissue-staining of seven subgroups of VA (AVM, LM, PG, GP, VeM, Hem, VM n.o.c.) and control tissues (Con) followed by digital image analysis with quantification of the staining signals.

## Materials and methods

### Tissues

A total of 40 human VA (Table 1) and five human control tissues (Table 2) were used for immunohistochemistry (IHC) and subsequent digital image analysis. All tested tissues were initially evaluated by a certified pathologist.

**Table 1:** Origin, type and anatomical location of VA tissues

No.	VA type	Localization	Gender
1	AVM	Upper lip	Male
2	AVM	Upper lip	Male
3	AVM	Parotid gland	Female
4	AVM	Face/nose	Male
5	AVM	Forehead	Female
6	AVM	Cheek and parotid gland	Male
7	AVM	Face	Male
8	LM	Upper lip/cheek	Female
9	LM	Tongue	Male
10	LM	Tongue	Female
11	LM	Perimandibular	Male
12	LM	Tongue	Male
13	VeM	Oro- and hypopharynx	Male
14	VeM	Cervical	Female
15	VeM	Orbita	Female
16	VeM	Sternocleidomastoid muscle	Female
17	Hem	Periorbital	Female
18	Hem	Sternocleidomastoid muscle	Female
19	Hem	Auricle	Female
20	Hem	n.s	Male
21	Hem	Cheek	Female
22	VM n.o.c	n.s	Male
23	VM n.o.c	Parotid gland	Female
24	VM n.o.c	Nasal cavity	Female
25	VM n.o.c	Endonasal	Female
26	VM n.o.c	Upper lip	Female
27	VM n.o.c	Face/lower lip	Female
28	VM n.o.c	Lower lip	Female
29	PG	Glomus caroticum	Male
30	PG	Glomus tympanicum	Female
31	PG	Cervical	Male
32	PG	Cervical	Male
33	PG	Interaortocaval	Female
34	PG	Glomus tympanicum	Female
35	GP	n.s	Female
36	GP	Vocal cord	Female
37	GP	n.s	Female
38	GP	n.s	Male
39	GP	Tongue	Female
40	GP	Tongue	Female

*AVM* arteriovenous malformation, *GP* pyogenic granuloma, *Hem* hemangioma, *LM* lymphatic malformation, *PG* paraganglioma, *VeM* venous malformation, *VM* (n.o.c.) vascular malformation (not otherwise classified), *n*.*s*. not specified

**Table 2:** Origin, type and anatomical location of control tissues

No.	VA type	Localization	Gender
1	Tumor-free nasal mucous membrane	Nasal	Male
2	Seborrheic wart	Cervical	Female
3	benign Mesenchymal tumor (fibroma)	Scalp	Female
4	Tumor free soft tissue	Preauricular	Male
5	Skin with subcutaneous fibrosis	Preauricular	Female

### Immunohistochemistry

Tissues were formalin-fixed and paraffin-embedded (FFPE). IHC was performed according to a standard laboratory protocol as reported elsewhere [12]. In short, 3 μm-thick FFPE slices were generated using a microtome followed by deparaffinization (Rotihistol; Carl Roth, Karlsruhe, Germany), alcohol incubation and heat treatment in 0.01 μmole/L trisodium citrate dihydrate buffer (92–95 °C, 20 min). Samples were blocked in normal goat serum and incubated with the specific primary antibody (Dako, Glostrup, Denmark). Antibodies directed against CD31 (dilution 1:50, clone JC70A, Dako), CD34 (dilution 1:50, clone QBEnd-10, Dako), PDPN (dilution 1:200, clone D2-40, Dako) and VIM (dilution 1:400, clone V9, Santa Cruz Biotechnology, Heidelberg, Germany) were mouse monoclonal. The anti-CLDN5 antibody (dilution 1:300, Zymed Laboratories, San Francisco, CA, USA) was rabbit polyclonal. Subsequently, the Universal Labelled (Strept)Avidin-Biotin2 System, HRP (Horseradish Peroxidase) (Dako) was deployed for secondary antibody binding followed by visualization of the DAB (3,3′-Diaminobenzidine) chromogen signal. Mouse IgG (Dako) was used as a negative control and did not exhibit any significant background (not shown). Samples were counterstained with Mayer’s Hemalaun solution (Merck, Darmstadt, Germany). Microscopic documentation of IHC-results was performed with the Axio Imager 2 microscope (AxioCam HRC camera, Carl Zeiss Microscopy GmbH, Jena, Germany) using the Application Suite software AxioVision Rel 4.8.

### Digital image analysis

**Fig. 1:**
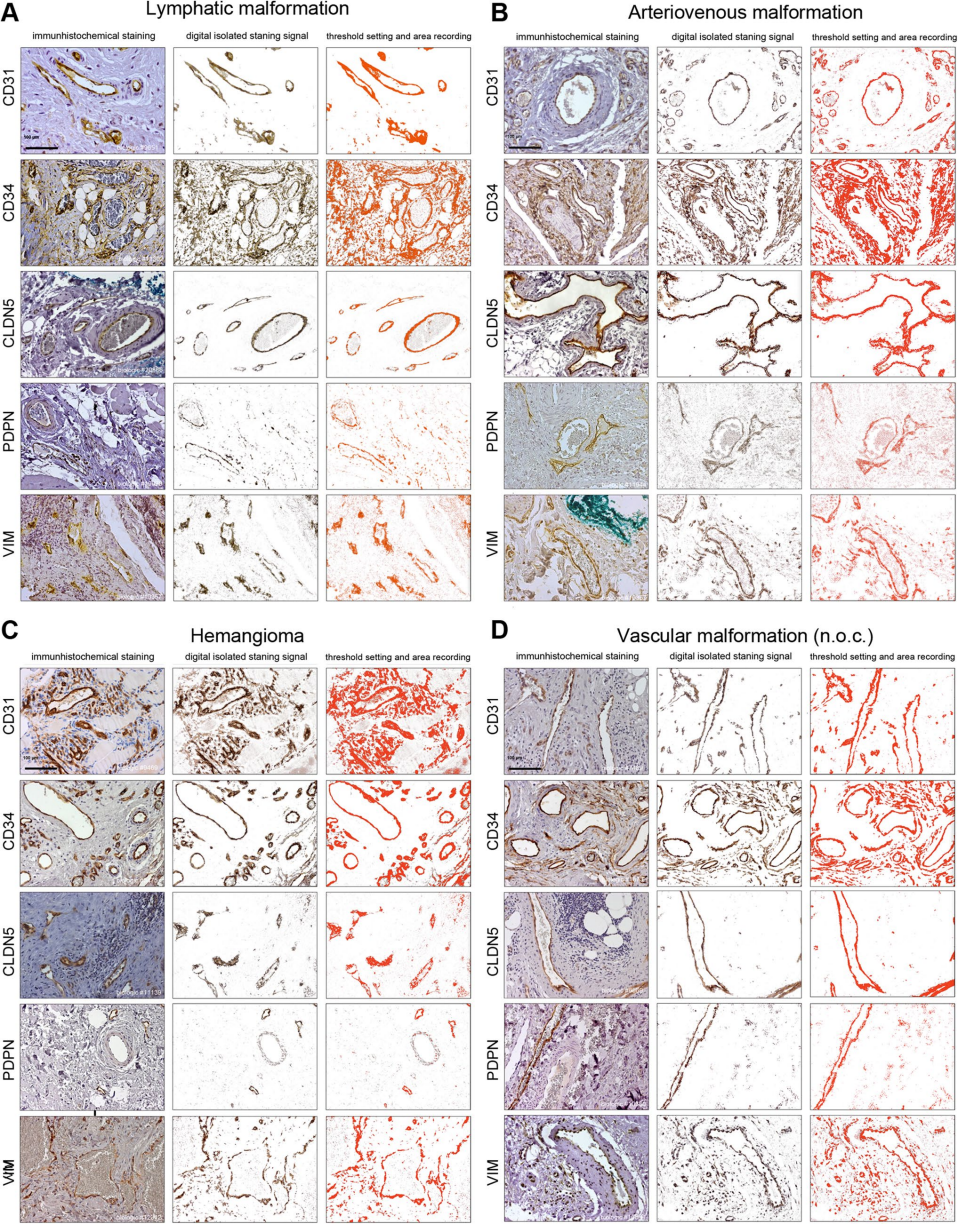

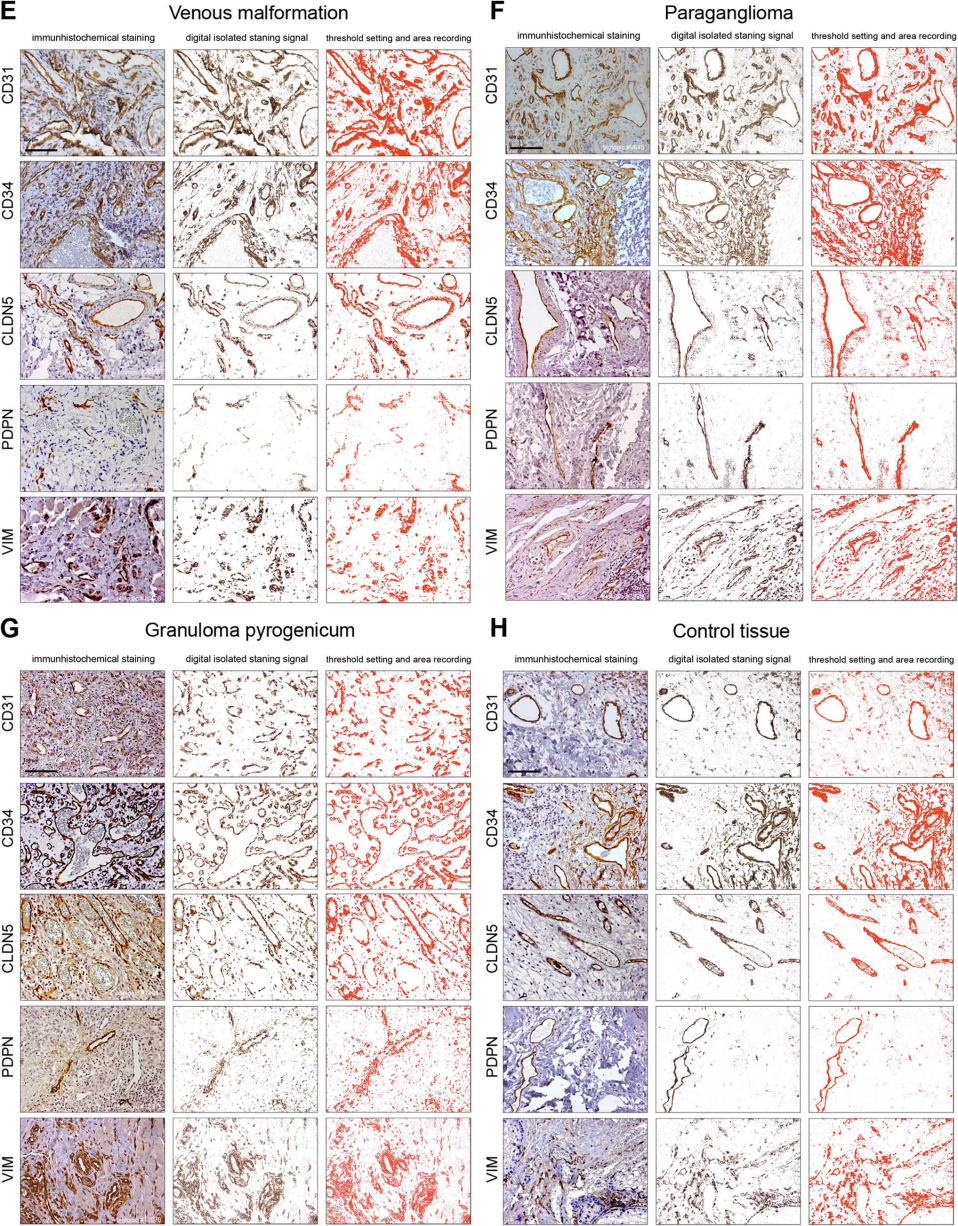
Detection of marker proteins in VA and capture of the signal. Representative immunohistochemical stainings of the tested marker proteins (CD31, CD34, CLDN5, PDPN, VIM) and subsequent working steps of digital image analysis for quantification are shown for **a** LM, **b** AVM, **c** Hem, **d** VM n.o.c., **e** VeM, **f** PG, **g** GP and **h** Con. Left column shows the original immunohistochemical image, middle column shows the digitally isolated staining signal (DAB, brown precipitate) using the ImageJ/Fiji-IHC-Toolbox (middle column) and right column the captured positive staining signal (right column, red signal) after subtraction of background staining wich was used for subsequent quantification. *AVM* arteriovenous malformation, *Con* control tissue, *GP* = pyogenic granuloma, *Hem* hemangioma, *LM* lymphatic malformation, *PG* paraganglioma, *VeM* venous malformation, *VM* (n.o.c.) vascular malformation (not otherwise classified). (20× magnification)

The open source program ImageJ/Fiji is a Java-based image processing program, developed at the National Institutes of Health and the Laboratory for Optical and Computational Instrumentation [13] which we used for scientific image analysis [5] together with the plugin *IHC toolbox* [14]. First, an immunohistochemically stained tissue section is captured and digitalized microscopically. All photographs were taken at 20 × magnification. Ten images were taken of each tissue section, in non-overlapping areas of VA or control tissues. To calculate the level of the specific DAB-signal based on the whole tissue in the photograph, the actual area of VA-tissue has to be calculated by subtracting the white (blank) areas which contain no tissue (e.g., lumina of vessels, artefacts). Figure 1 depicts exemplary immunohistochemical images for all protein markers and tissues followed by digital capture and isolation of the specific signals (red).

### Statistical analysis

VA are counted among the rare diseases, which explains why a limited number of tissue samples was used. For each staining, ten independent non-overlapping areas were evaluated for a given tissue sample and the mean value and SEM were calculated for each type of VA.

**Fig. 2:**
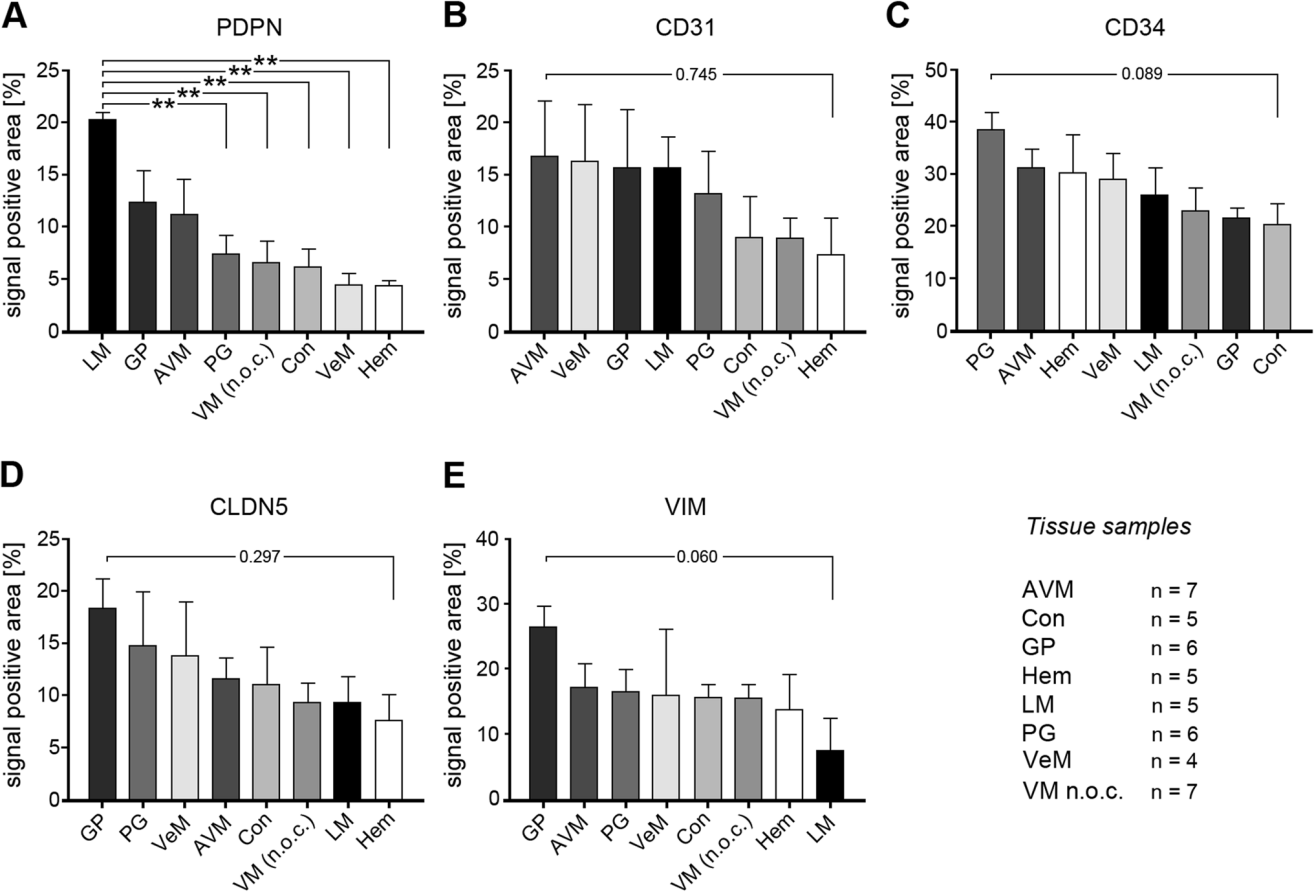
Expression levels of marker proteins in VA tissues. Depicted is the mean (± SEM) of the respective quantified signal values for **a** PDPN, **b** CD31, **c** CD34, **d** CLDN5, and **e** VIM for all 8 tested VA types. *AVM* arteriovenous malformation, *Con* control tissue, *GP* pyogenic granuloma, *Hem* hemangioma, *LM* lymphatic malformation, *PG* paraganglioma, *VeM* venous malformation, *VM* (n.o.c.) vascular malformation (not otherwise classified). (**p < 0.01)

**Fig. 3:**
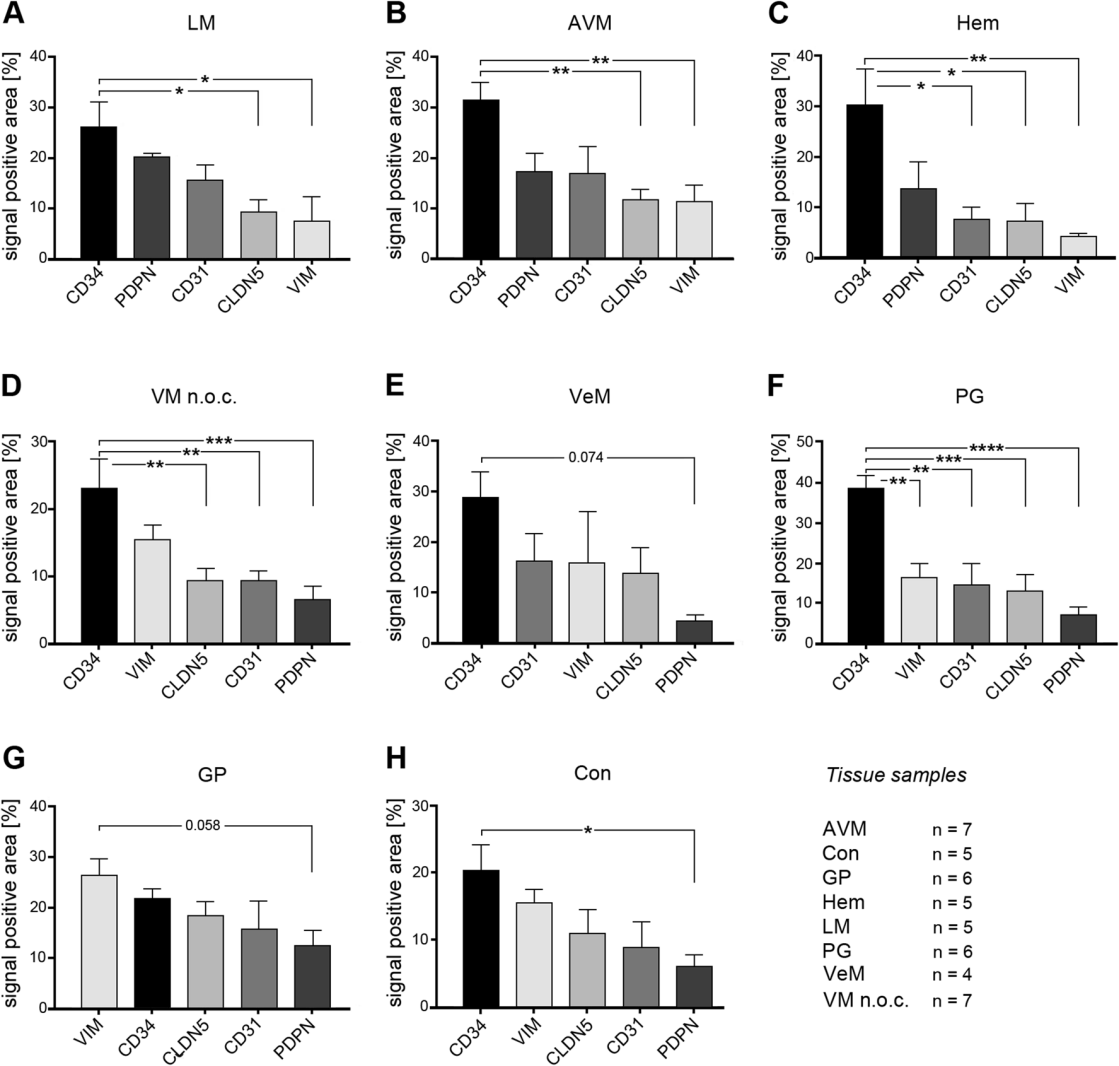
Comparison of marker protein expression levels within each tested VA. Shown are the relative expression levels of CD31, CD34, CLDN5, PDPN and VIM in **a** LM, **b** AVM, **c** Hem, **d** VM n.o.c., **e** VeM, **f** PG, **g** GP and **h** Con. *AVM* arteriovenous malformation, *Con* control tissue, *GP* pyogenic granuloma, *Hem* hemangioma, *LM* = lymphatic malformation, *PG* paraganglioma, *VeM* venous malformation, *VM* (n.o.c.) vascular malformation (not otherwise classified). (*p < 0.05, **p < 0.01, ***p < 0.001, ****p < 0.0001)

**Fig. 4:**
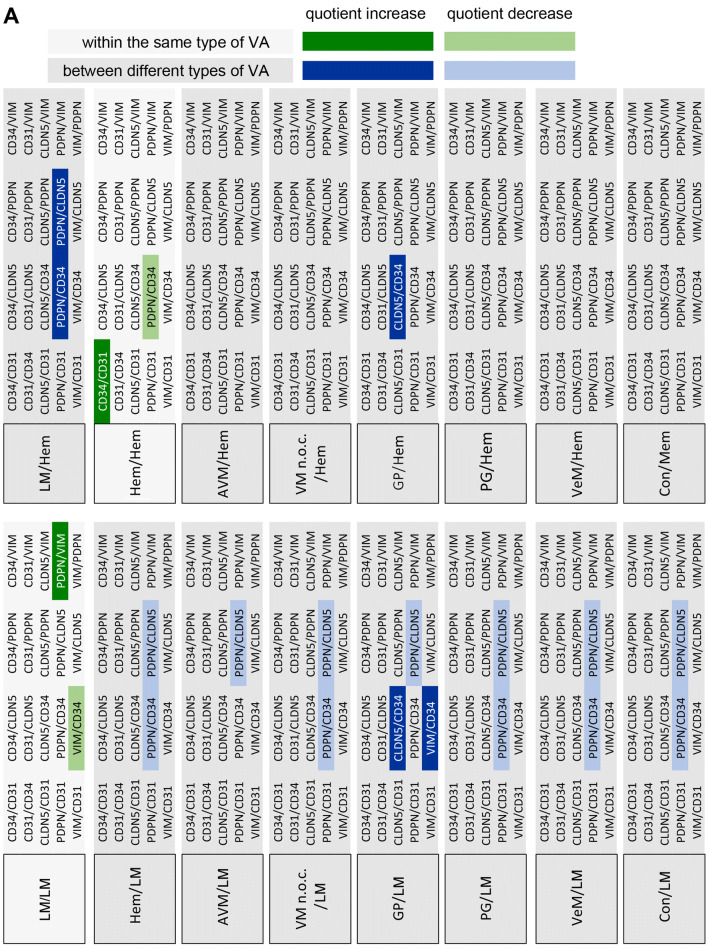

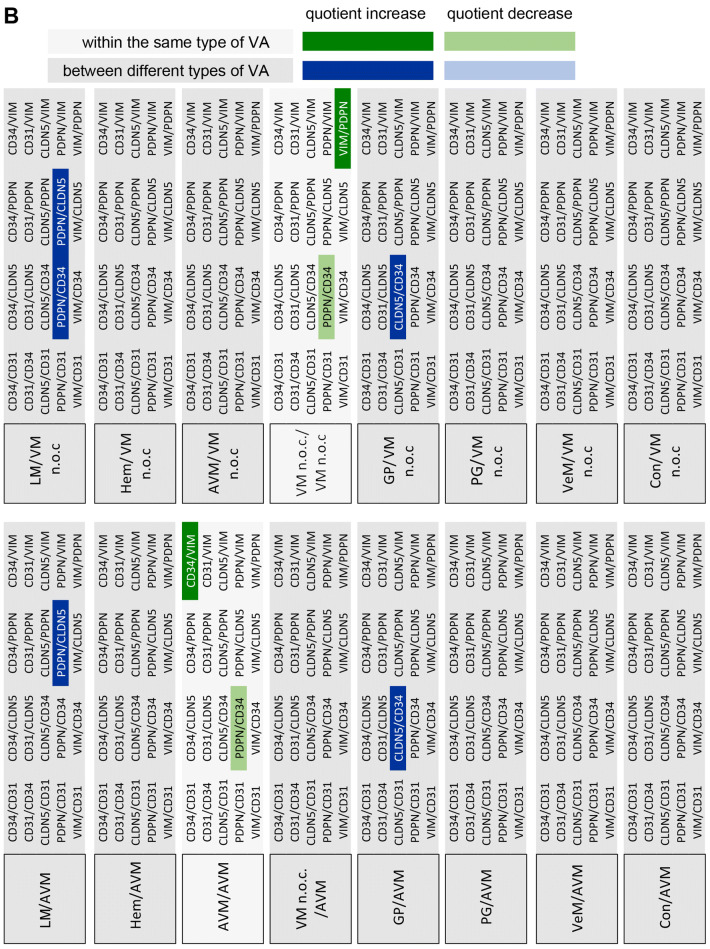

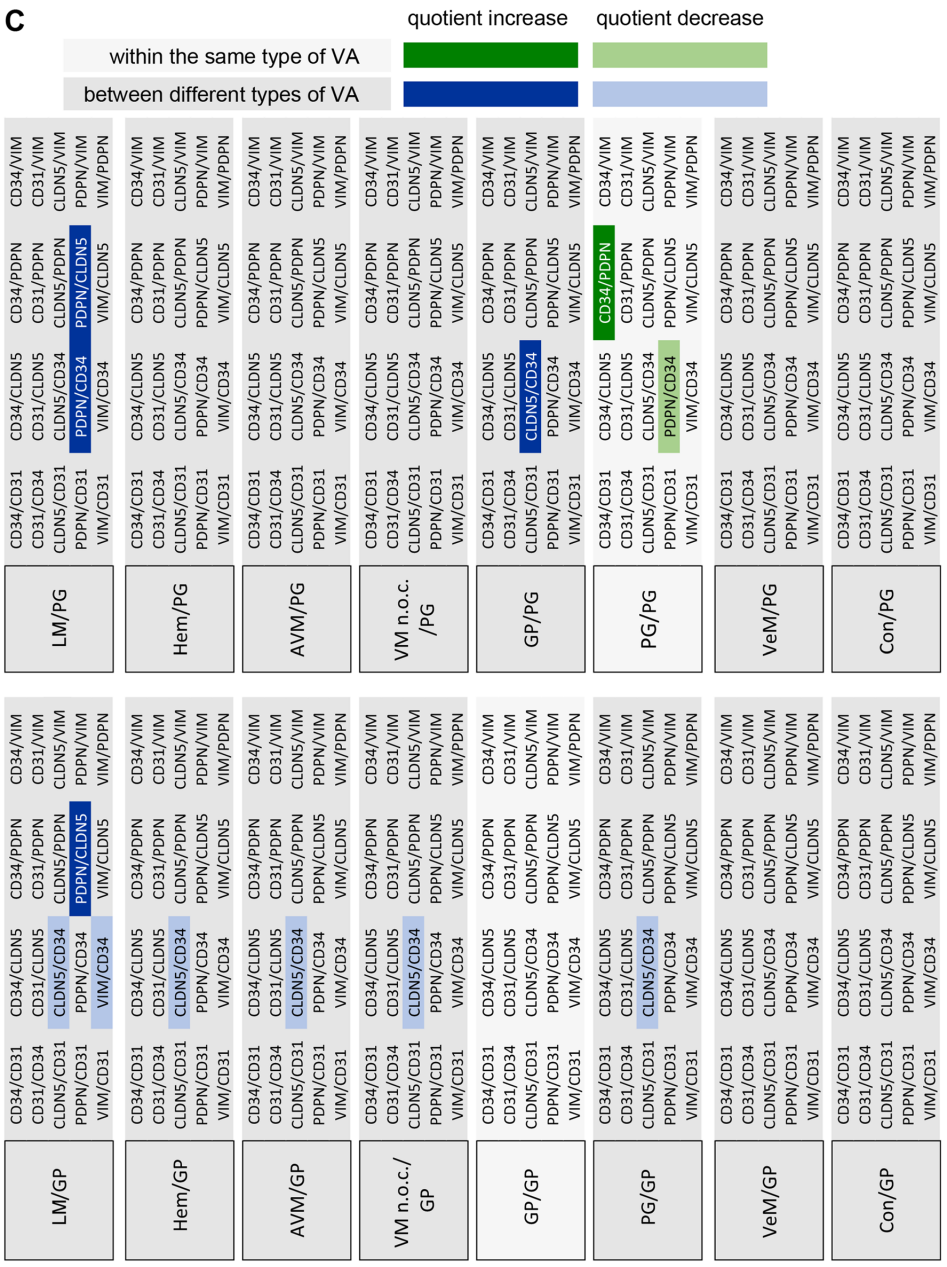

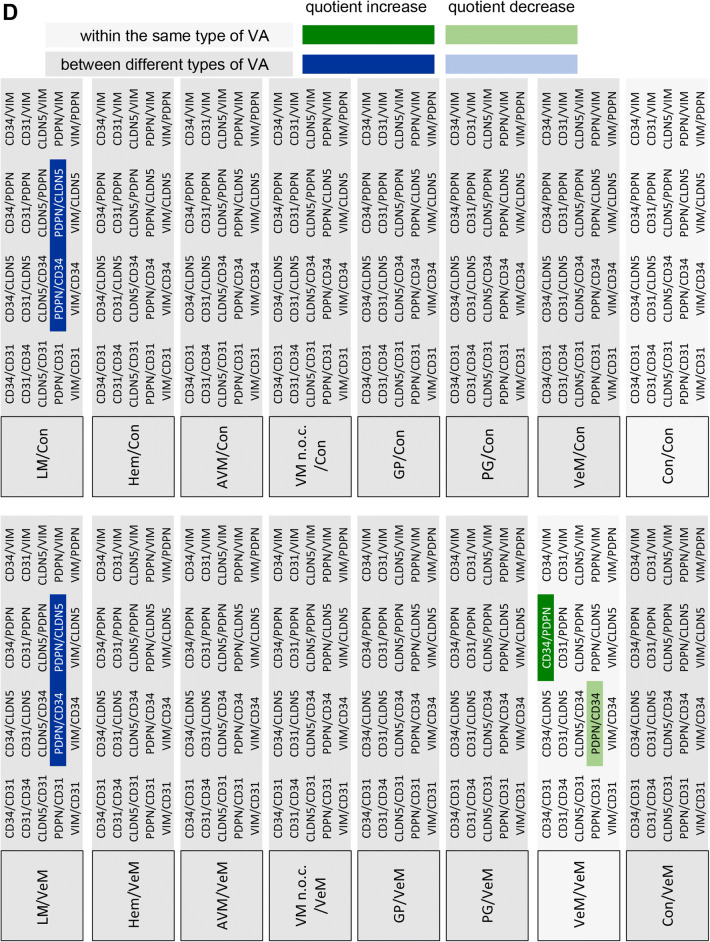
Matrix of all marker signal quotients calculated for the tested tissues. Depicted is a comparison of the 20 different marker signal quotients between the tested 8 tissues. **a**–**d** Only significantly different marker signal values (*p* < 0.05) were considered as relevant to be used for tissue stratification (highlighted). For example, when comparing GP and LM (**a**, GP/LM) the signal marker quotient CLDN5/CD34 is found to be significantly different between both tissue types (= significantly higher in GP than in LM, highlighted in dark blue). *AVM* arteriovenous malformation, *Con* control tissue, *GP* pyogenic granuloma, *Hem* hemangioma, *LM* lymphatic malformation, *PG* paraganglioma, *VeM* venous malformation, *VM* (n.o.c.) vascular malformation (not otherwise classified)

For statistical analysis of the expression of the different markers in the respective VA tissues three approaches were chosen. First, the expression levels of the individual candidate proteins between the different VAs were analyzed (Fig. 2). Second, the expression levels of all candidate proteins within a specific VA were compared (Fig. 3). Finally, quotients between the mean values of 2 candidate proteins each within a VA and between the different VAs were compared (Fig. 4). The consideration of the quotients ensures that the individual quotients of a single tissue section or each patient were taken into account. For each marker protein staining, the quantification of ten independent, non-overlapping areas was calculated. The mean value for the respective VA type (*n* = 4 to 7) was calculated from these. An ordinary one-way ANOVA test was used for all statistical calculations followed by correction of Turkey’s multiple comparison test. A level of significance of 0.05 was chosen, *p*-values under 0.05 were seen as statistically significant. The GraphPad Prism 7 software was used.

## Results

### Comparison of single candidate-marker expression levels between different tissues

Different expression levels of single marker proteins were seen between the different tested tissues (Fig. 2). However, only for PDPN differences in expression reached significance (*p* < 0.01), as shown in Fig. 2a when comparing LM with PG, VM (n.o.c.), Con, VeM and Hem. No significant differences in CD31 signal values could be detected between the 8 tested tissues (Fig. 2b). Similar CD34 expression levels (20–30%) were detected in most tested tissue types, whereas on average markedly higher (38.7%) expression levels, although not reaching significance, were seen in PG (Fig. 2c). 18.5% of GP tissue areas were CLDN5-positive representing the highest level but did not reach significance compared with the other tissues (Fig. 2d). Similarly, VIM stained a total of 26.5% of GP tissue areas, whereas only 8% VIM-positive areas were seen in LM. However, these differences where not significant (*p * = 0.06) (Fig. 2e).

### Relative expression of candidate marker proteins

Staining of the 5 candidate markers within each tissue type was evaluated to assess their relative expression (Fig. 3). Except for GP, CD34 was the most predominant marker in all tested tissues. With on average 26%, CD34 staining was significantly higher in LM-tissues than CLDN5 (9.4%) and VIM (7.6%) (*p* < 0.05). (Fig. 3a). AVM tissues expressed CD34 at 31.4%, which was significantly higher (*p* < 0.01) than the CLDN5 and VIM signals (11%) (Fig. 3b). CD34 was expressed around 30% in Hem tissues which was significantly higher than the CD31, CLDN5 (around 7%, *p* < 0.05) and VIM (5%, *p* < 0.01) staining (Fig. 3c). Staining an average area of about 23.3%, CD34 exhibited the highest expression level in VM n.o.c.. CD34 values were significantly higher than PDPN (6.7%, *p* < 0.001), CLDN5 and CD31 (9%, *p* < 0.01) levels (Fig. 3d). In PG, with 38%, the endothelial marker CD34 exhibited significantly higher expression levels than VIM (16.5%, p < 0.01), CD31 (13%, *p* < 0.01), CLDN5 (14.8%, *p* < 0.001) and PDPN (7.4%, *p* < 0.0001) (Fig. 3f) and also was higher than in any other tested tissue. 20.5% of the control tissue area expressed CD34 which was significantly higher (*p* < 0.05) than PDPN expression levels (6.1%) (Fig. 3h). No statistical significant differences between the five tested markers were seen in VeM and GP (Fig. 3e, g).

### Using marker signal quotients to improve stratification of the different VA tissues

**Fig. 5:**
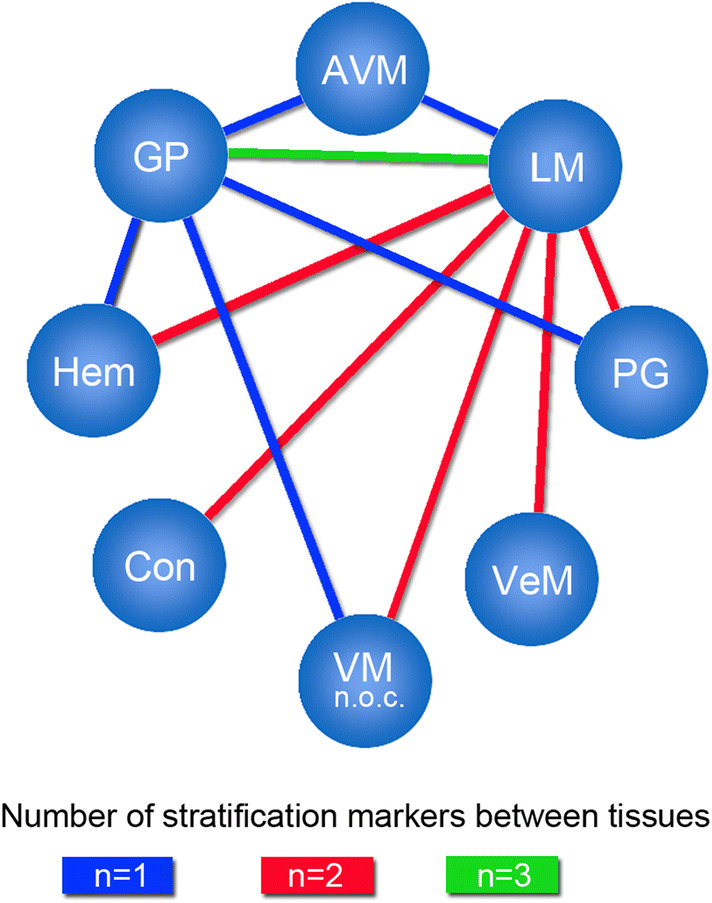
Level of VA tissue stratification achieved by using signal quotients from 5 marker proteins. Depicted is a graphical comparison of the tested 8 tissues deploying the identified marker signal quotients. Connecting lines between the tissues are indicating the presence of at least one significant marker signal quotient between the two connected tissue types that can be used for stratification of both tissue types. A connecting blue line indicates the presence of one, a red line of two and a green line of three significant marker signal quotients between the respective tissues. *AVM* arteriovenous malformation, *Con* control tissue, *GP* pyogenic granuloma, *Hem* hemangioma, *LM* lymphatic malformation, *PG* paraganglioma, *VeM* venous malformation, *VM* (n.o.c.) vascular malformation (not otherwise classified)

Since the relative expression of the 5 marker proteins within a specific tissue type appeared variable among the different tissue types, the quotients of marker signal values within a given tissue was compared with the corresponding quotients of marker signal values from different tissue types. Only significantly different quotients (*p* < 0.05) were considered as markers for stratification of two distinct tissue types (Fig. 4). Figure 5 depicts the level of stratification achieved for the 8 tissue types when using the significantly different quotients as depicted in Fig. 4. LM was the only VA that could be fully distinguished from all other 7 tested tissue types. GP could be separated from 5 VA tissues (LM, Hem, VM, PG, AVM), whereas Hem, PG, VM and AVM all could be distinguished from LM and GP tissues. VeM and Con only appeared different from LM tissues based on significantly different signal marker quotients (Fig. 5).

## Discussion

The 2014 revised ISSVA (International Society for the Study of Vascular Anomalies) classification provides a standardized nomenclature for VA [1]. This nomenclature is mainly based on the classification according to Mulliken and Glowacki [3]. Making an accurate diagnosis, however, is frequently complicated due to the heterogeneous clinical and histopathological appearance of VA. Due to these difficulties, the initial diagnosis is reported incorrectly in up to 69% of cases. Similarly, to complicate it even more, incorrect terminology is frequently used for description of VA [15]. Making an exact diagnosis is clearly a prerequisite to select adequate therapeutic approaches for the treatment of VA. The ISSVA classification follows clinical criteria as well as histopathological findings. The latter have proven their importance for a more precise classification of VA with regard to subgroups such as vascular malformations and vascular tumors [16, 17]. In addition, not only the correct diagnosis but also the course of VA development has to be taken into account. Furthermore, immunohistochemical markers or candidate genes and proteins in some instances are useful to learn more about the expression and possible functional impairment of specific genes in VA [4]. For example, mutations in the PIK3CA gene were found associated with the development of venous and lymphatic malformations or syndromes including these types of VA [18]. In the present study, the expression of vascular markers was investigated on a selection of clinically most relevant VA. Here, quantitative digital image analysis was evaluated as an instrument for VA stratification to support the histopathological diagnosis. Digital image analysis of VA could contribute to an improved subclassification of VA and has demonstrated its suitability as a potential tool for a more exact and less biased diagnosis of VA, which potentially could be helpful in therapeutic decision making. Not unexpected, the use of the well-established endothelial markers CD34 and CD31 exhibited major immune reactivity of vascular endothelia in all tested tissues. The usefulness of PDPN to distinguish VA of lymphatic origin from other malformations could be demonstrated. This underlines the reliability of PDPN as a lymphatic marker that allows differentiation of VA. The detection of CLDN5 in the endothelium of VA supports its previously postulated role in differentiation and maintenance of vascular structures. However, it also became obvious that diagnosis of VA should not be restricted to one or a few immunohistochemical markers. Rather it appears necessary to create an “expression profile” for each tissue, based on characteristic protein expression-patterns of the markers. In this study we were able to demonstrate successful stratification of VA tissues by deploying five marker proteins. However, further studies including a higher number of VA tissues and additional marker proteins should follow, which should allow for complete stratification of all VA. Digital image analysis appears to be a promising tool for measuring differences in the expression of markers such as those used in VA tissues. Results of this study support the usefulness of digital analysis for classification of the heterogeneous group of VA. However, it should be emphasized, that its potential application cannot replace an experienced pathologist but rather assist in the diagnosis. In addition it is important to note, that immunohistochemical results have to be considered in the context of the overall clinical picture. There are still many inconsistencies among histopathological and clinical classifications of VA which can lead to false diagnoses negatively affecting therapeutic decision making. With regards to future diagnostic methods, it can be expected that the use of digital image analysis will continue to increase. Therefore, establishment of digital methods for histological evaluation of tissue samples could be helpful. Strongly related to digital image analysis is the new field of artificial intelligence and deep (machine) learning deploying convolutional neural networks. These computer based techniques can be applied for image recognition. For this, such programs have to be fed with digital information such as stained histopathological specimens on a much higher scale. Specific algorithms would then allow for stratification of different tissue types as those found in the different VA subgroups. In this context, a recent study by Skrede and colleagues deployed deep (machine) learning to predict colorectal cancer outcome. For this, they used 12,000,000 standard hematoxylin & eosin stained tumor tissue sections to train 10 convolutional neural networks. Here they could stratify stage II and III patients into more exact prognostic groups which potentially will help to deploy more personalized therapy schemes [19]. Furthermore, several studies deployed deep learning to evaluate retinal diseases such as diabetic retinopathy and macular degeneration by using fundus images of the eyes [20, 21]. Our study, therefore, serves as a paradigm for the potential diagnostic benefit of computer based digital analysis techniques for the stratification of VA. Digital image analysis as performed in our study and more so deep (machine) learning, therefore, represent promising supportive tools not only for the stratification of malignant tumors but also of morphologically highly complex tissues such as VA.
